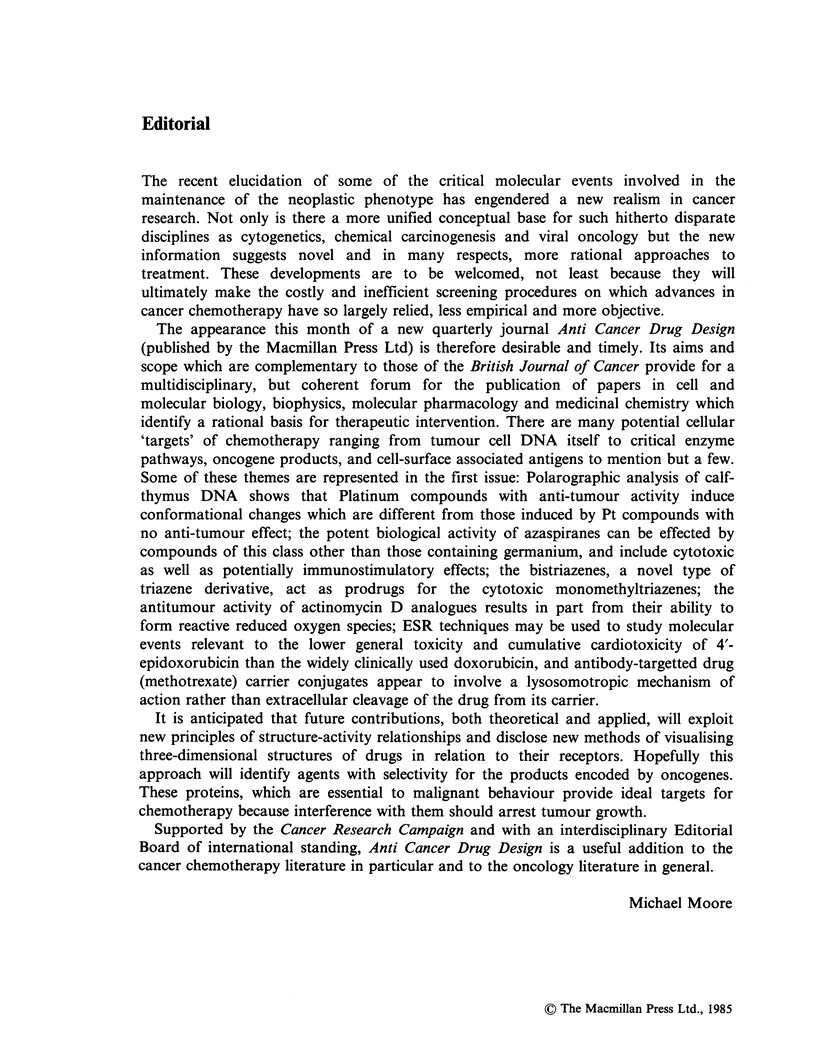# Anti-Cancer Drug Design

**Published:** 1985-11

**Authors:** Michael Moore


### Editorial

The recent elucidation of some of the critical molecular events involved in the maintenance of the neoplastic phenotype has engendered a new realism in cancer research. Not only is there a more unified conceptual base for such hitherto disparate disciplines as cytogenetics, chemical carcinogenesis and viral oncology but the new information suggests novel and in many respects, more rational approaches to treatment. These developments are to be welcomed, not least because they will ultimately make the costly and inefficient screening procedures on which advances in cancer chemotherapy have so largely relied, less empirical and more objective.

The appearance this month of a new quarterly journal *Anti Cancer Drug Design* (published by the Macmillan Press Ltd) is therefore desirable and timely. Its aims and scope which are complementary to those of the *British Journal of Cancer* provide for a multidisciplinary, but coherent forum for the publication of papers in cell and molecular biology, biophysics, molecular pharmacology and medicinal chemistry which identify a rational basis for therapeutic intervention. There are many potential cellular 'targets' of chemotherapy ranging from tumour cell DNA itself to critical enzyme pathways, oncogene products, and cell-surface associated antigens to mention but a few. Some of these themes are represented in the first issue: Polarographic analysis of calf-thymus DNA shows that Platinum compounds with anti-tumour activity induce conformational changes which are different from those induced by Pt compounds with no anti-tumour effect; the potent biological activity of azaspiranes can be effected by compounds of this class other than those containing germanium, and include cytotoxic as well as potentially immunostimulatory effects; the bistriazenes, a novel type of triazene derivative, act as prodrugs for the cytotoxic monomethyltriazenes; the antitumour activity of actinomycin D analogues results in part from their ability to form reactive reduced oxygen species; ESR techniques may be used to study molecular events relevant to the lower general toxicity and cumulative cardiotoxicity of 4′-epidoxorubicin than the widely clinically used doxorubicin, and antibody-targetted drug (methotrexate) carrier conjugates appear to involve a lysosomotropic mechanism of action rather than extracellular cleavage of the drug from its carrier.

It is anticipated that future contributions, both theoretical and applied, will exploit new principles of structure-activity relationships and disclose new methods of visualising three-dimensional structures of drugs in relation to their receptors. Hopefully this approach will identify agents with selectivity for the products encoded by oncogenes. These proteins, which are essential to malignant behaviour provide ideal targets for chemotherapy because interference with them should arrest tumour growth.

Supported by the *Cancer Research Campaign* and with an interdisciplinary Editorial Board of international standing, *Anti Cancer Drug Design* is a useful addition to the cancer chemotherapy literature in particular and to the oncology literature in general.

Michael Moore

© The Macmillan Press Ltd., 1985